# Supplementary material for: QTL Mapping for Resistance to Cankers Induced by Pseudomonas syringae pv. actinidiae (Psa) in a Tetraploid Actinidia chinensis Kiwifruit Population
Source: Pathogens. 2020 Nov 20;9(11):967. doi: 10.3390/pathogens9110967 (PMC7709049; doi:10.3390/pathogens9110967)
Supplement: Supplementary file 1 [file pathogens-09-00967-s001.zip › Supplementary material_revised_FINAL_Revised.pptx]

## Slide 1
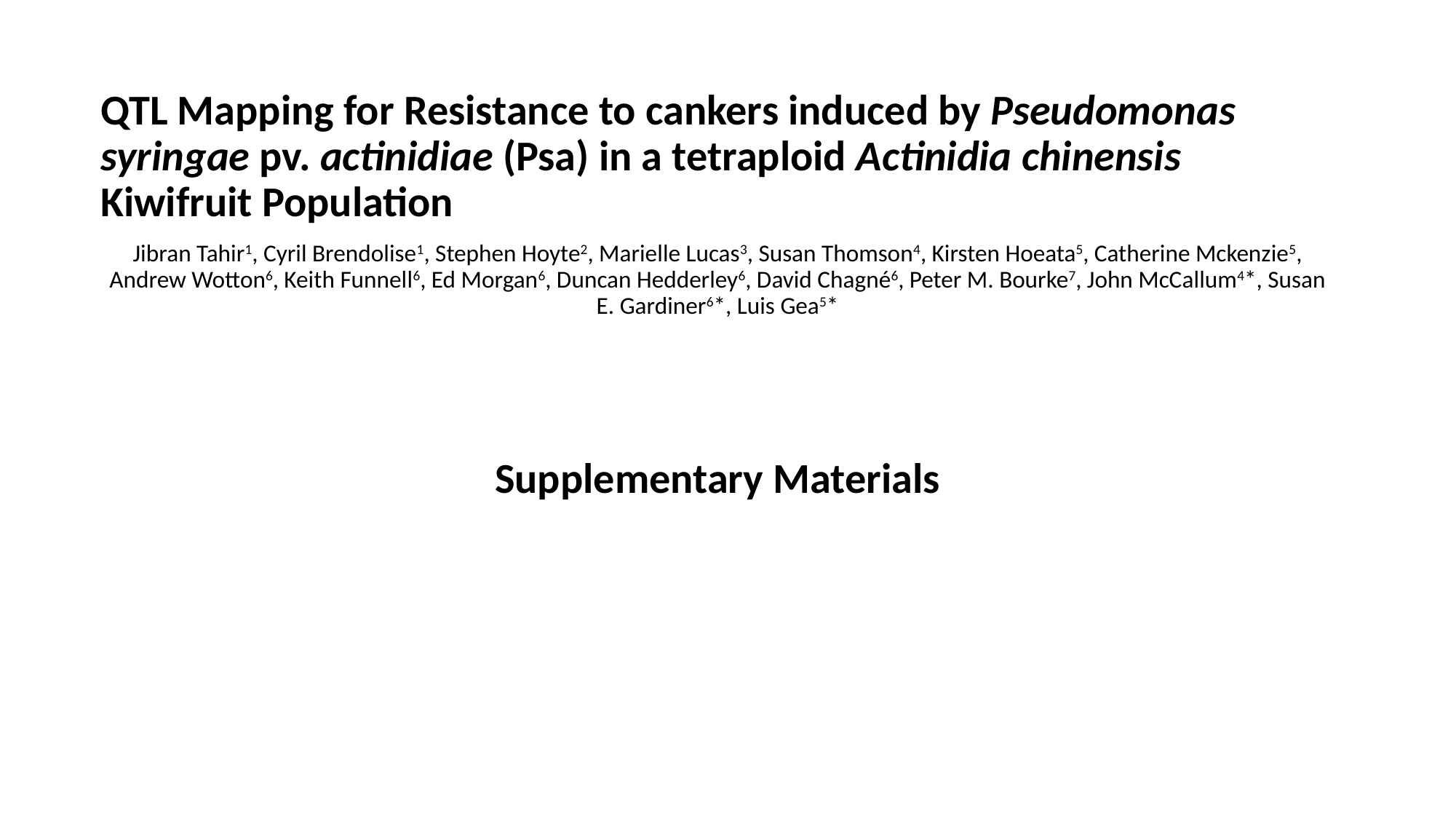

QTL Mapping for Resistance to cankers induced by Pseudomonas syringae pv. actinidiae (Psa) in a tetraploid Actinidia chinensis Kiwifruit Population
Jibran Tahir1, Cyril Brendolise1, Stephen Hoyte2, Marielle Lucas3, Susan Thomson4, Kirsten Hoeata5, Catherine Mckenzie5, Andrew Wotton6, Keith Funnell6, Ed Morgan6, Duncan Hedderley6, David Chagné6, Peter M. Bourke7, John McCallum4*, Susan E. Gardiner6*, Luis Gea5*
Supplementary Materials

## Slide 2
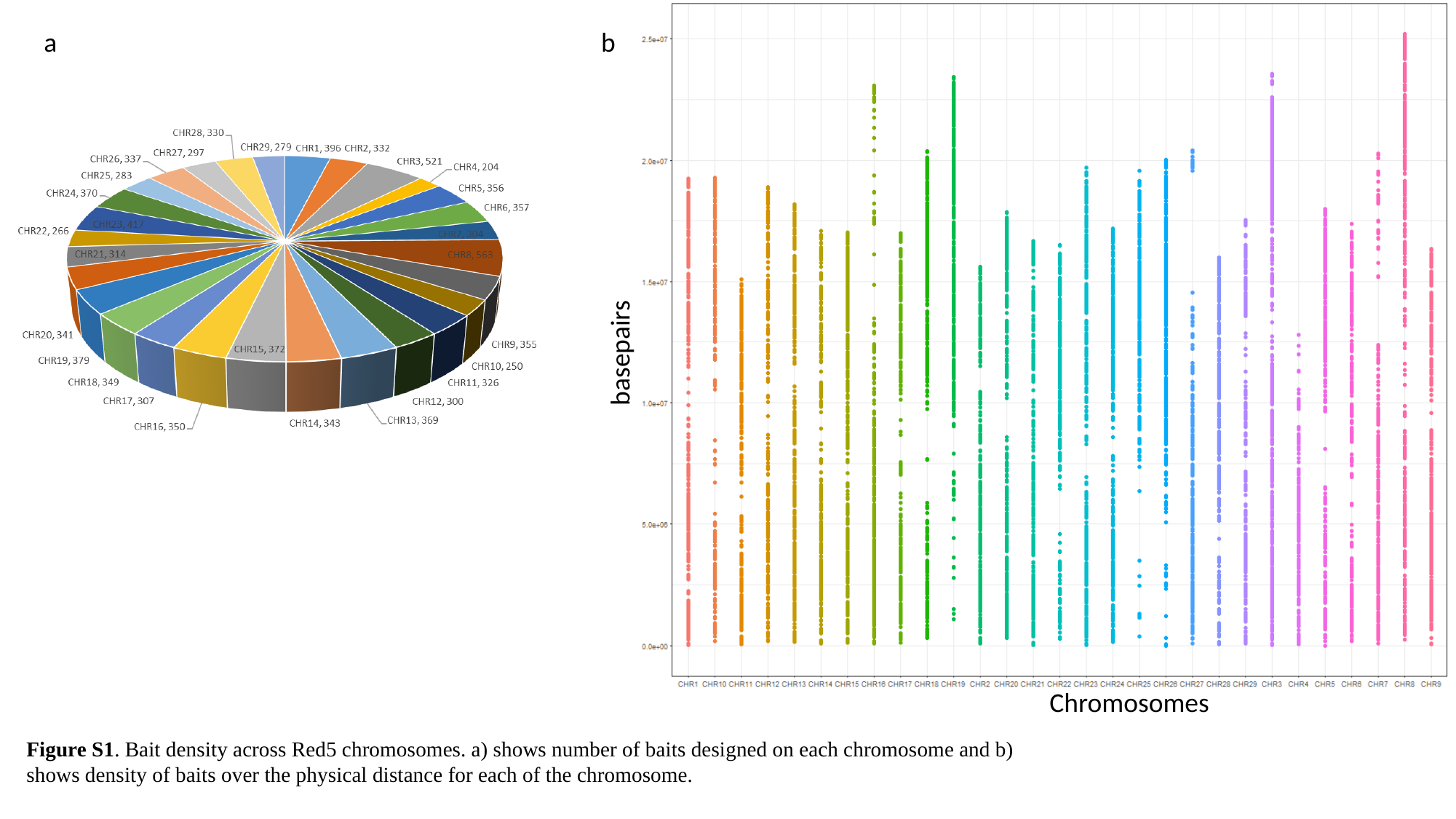

a
b
basepairs
Chromosomes
Figure S1. Bait density across Red5 chromosomes. a) shows number of baits designed on each chromosome and b) shows density of baits over the physical distance for each of the chromosome.

## Slide 3
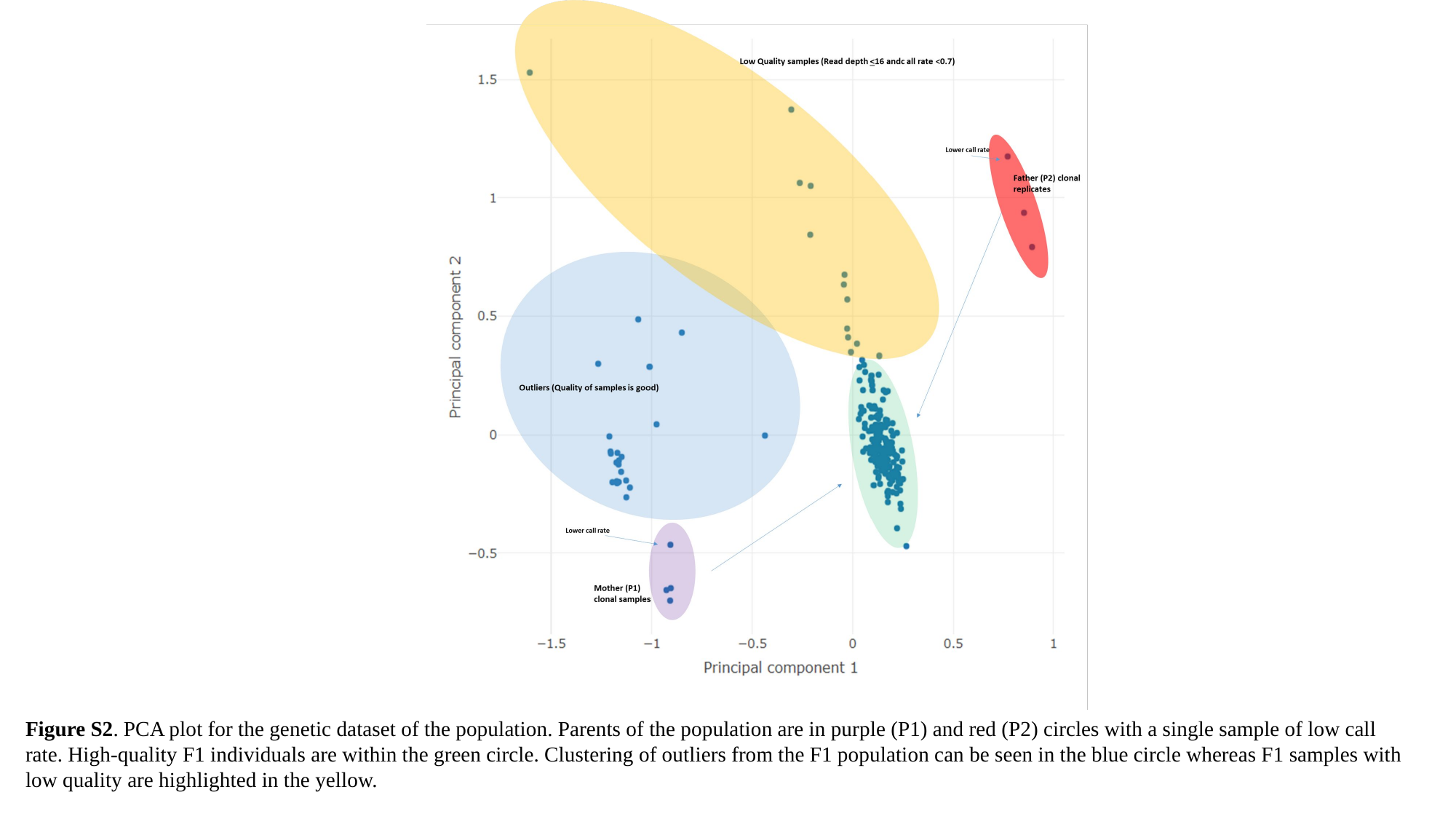

Figure S2. PCA plot for the genetic dataset of the population. Parents of the population are in purple (P1) and red (P2) circles with a single sample of low call rate. High-quality F1 individuals are within the green circle. Clustering of outliers from the F1 population can be seen in the blue circle whereas F1 samples with low quality are highlighted in the yellow.

## Slide 4
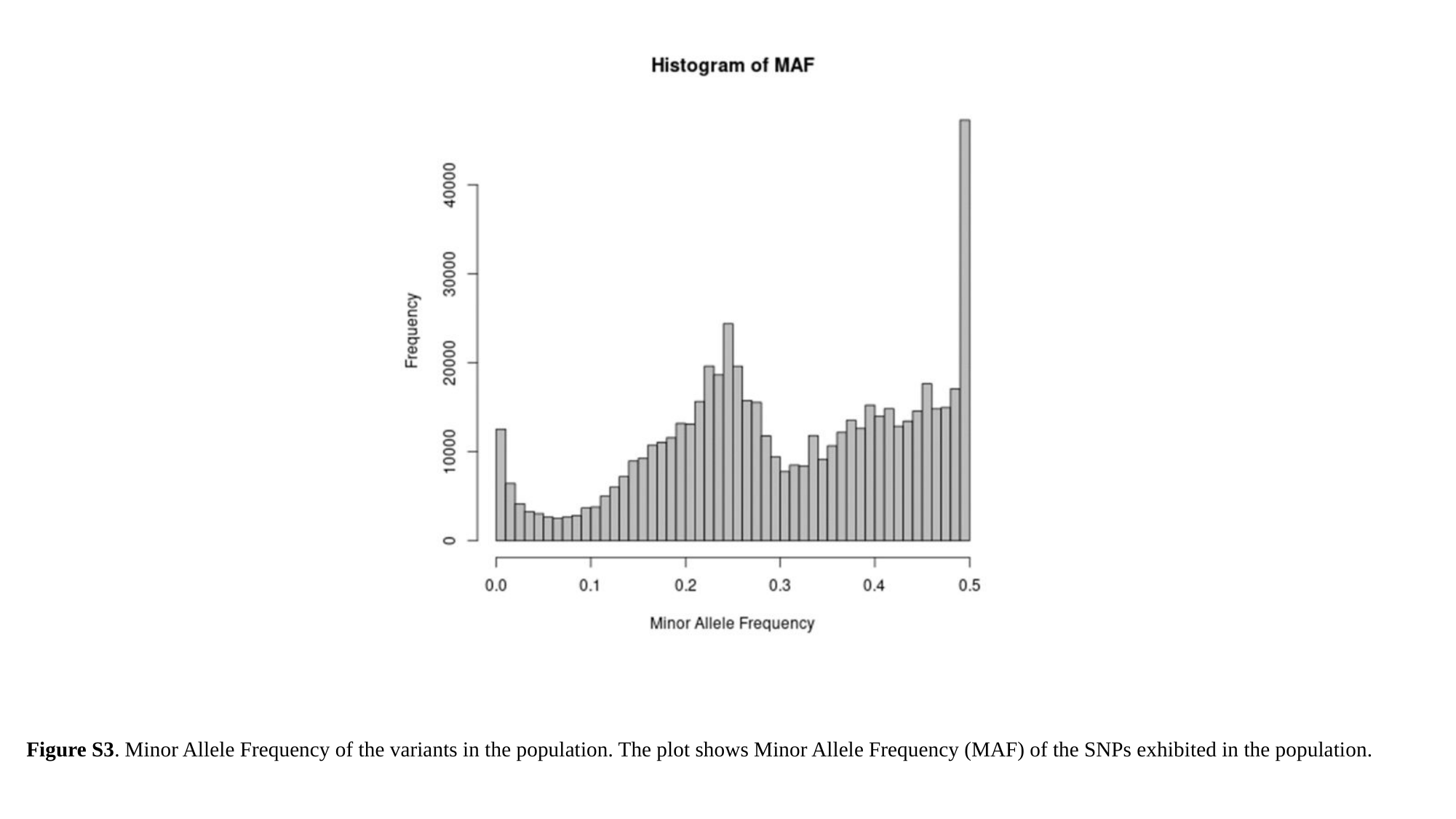

Figure S3. Minor Allele Frequency of the variants in the population. The plot shows Minor Allele Frequency (MAF) of the SNPs exhibited in the population.

## Slide 5
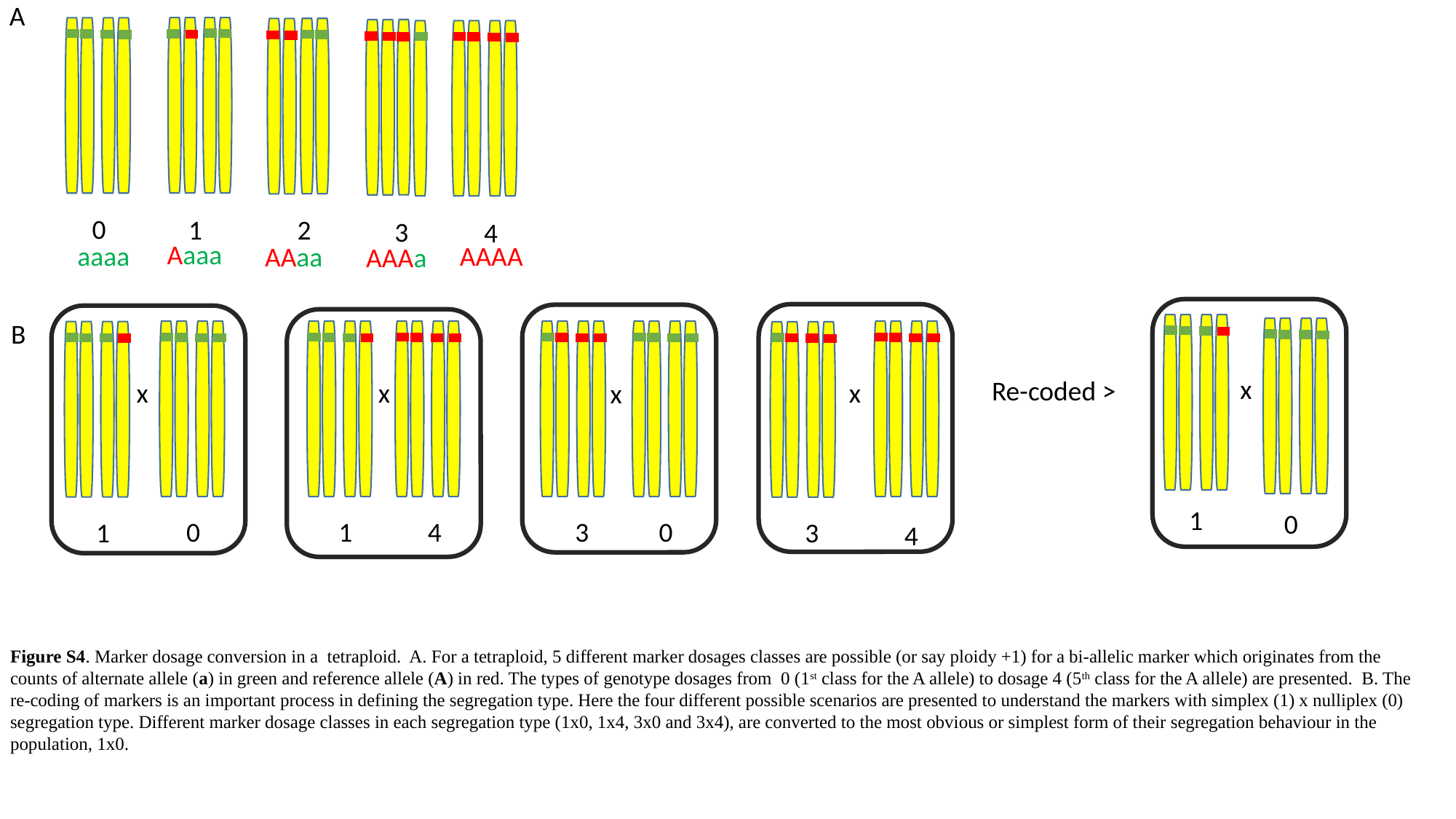

A
0
1
2
3
4
Aaaa
aaaa
AAAA
AAaa
AAAa
B
x
Re-coded >
x
x
x
x
1
0
3
4
0
0
1
3
1
4
Figure S4. Marker dosage conversion in a tetraploid. A. For a tetraploid, 5 different marker dosages classes are possible (or say ploidy +1) for a bi-allelic marker which originates from the counts of alternate allele (a) in green and reference allele (A) in red. The types of genotype dosages from 0 (1st class for the A allele) to dosage 4 (5th class for the A allele) are presented. B. The re-coding of markers is an important process in defining the segregation type. Here the four different possible scenarios are presented to understand the markers with simplex (1) x nulliplex (0) segregation type. Different marker dosage classes in each segregation type (1x0, 1x4, 3x0 and 3x4), are converted to the most obvious or simplest form of their segregation behaviour in the population, 1x0.

## Slide 6
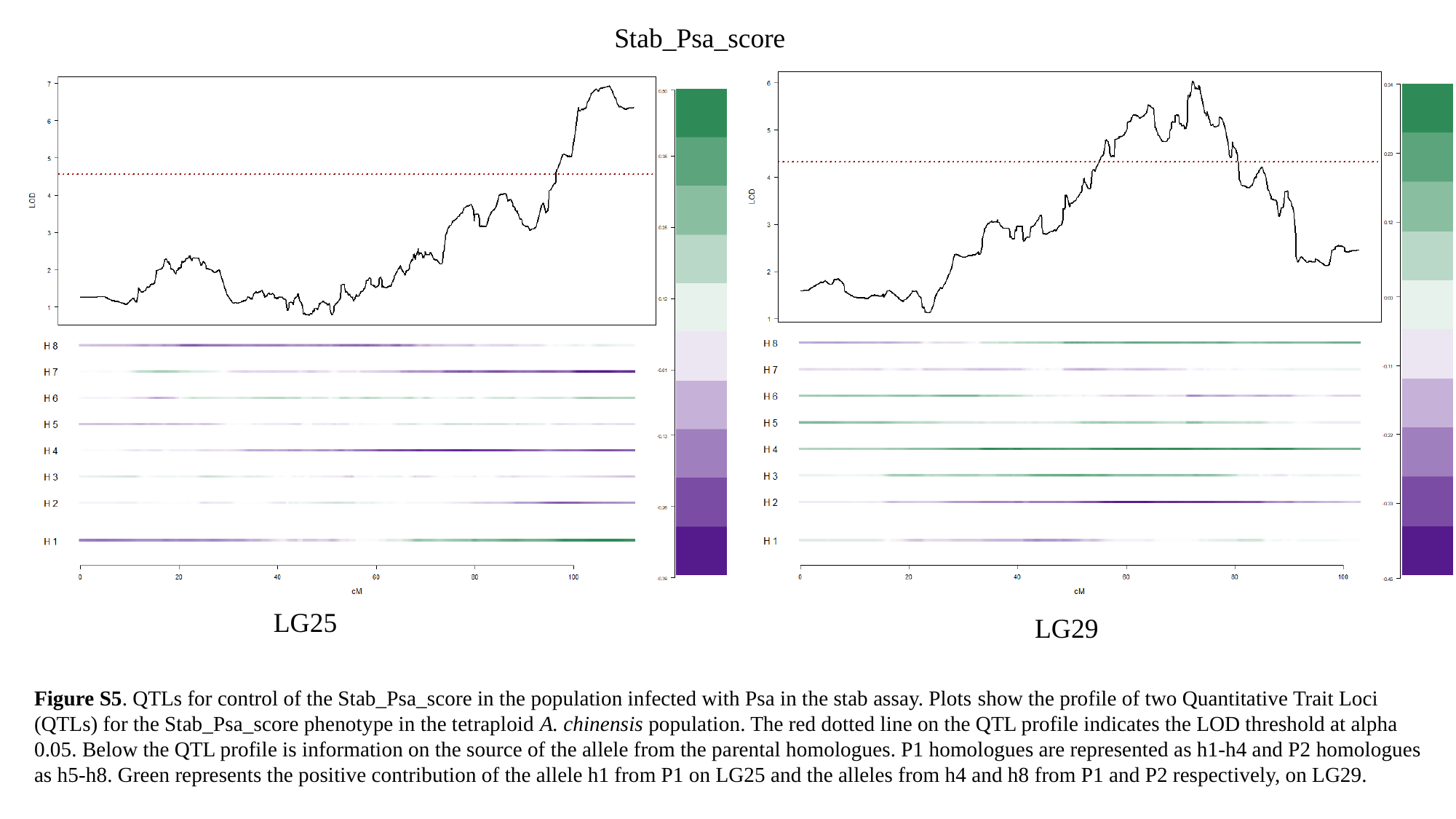

Stab_Psa_score
LG25
LG29
Figure S5. QTLs for control of the Stab_Psa_score in the population infected with Psa in the stab assay. Plots show the profile of two Quantitative Trait Loci (QTLs) for the Stab_Psa_score phenotype in the tetraploid A. chinensis population. The red dotted line on the QTL profile indicates the LOD threshold at alpha 0.05. Below the QTL profile is information on the source of the allele from the parental homologues. P1 homologues are represented as h1-h4 and P2 homologues as h5-h8. Green represents the positive contribution of the allele h1 from P1 on LG25 and the alleles from h4 and h8 from P1 and P2 respectively, on LG29.

## Slide 7
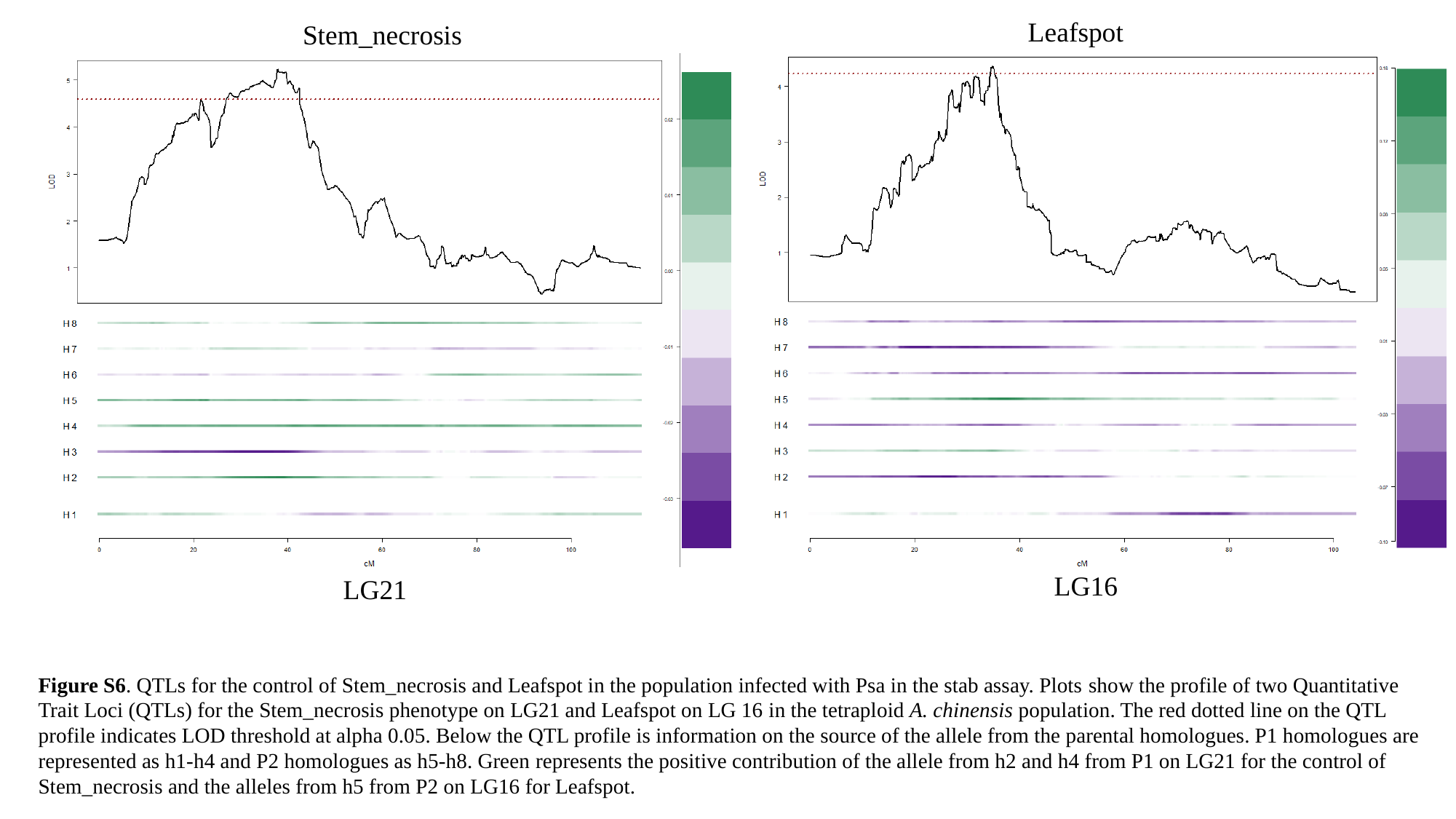

Leafspot
Stem_necrosis
LG16
LG21
Figure S6. QTLs for the control of Stem_necrosis and Leafspot in the population infected with Psa in the stab assay. Plots show the profile of two Quantitative Trait Loci (QTLs) for the Stem_necrosis phenotype on LG21 and Leafspot on LG 16 in the tetraploid A. chinensis population. The red dotted line on the QTL profile indicates LOD threshold at alpha 0.05. Below the QTL profile is information on the source of the allele from the parental homologues. P1 homologues are represented as h1-h4 and P2 homologues as h5-h8. Green represents the positive contribution of the allele from h2 and h4 from P1 on LG21 for the control of Stem_necrosis and the alleles from h5 from P2 on LG16 for Leafspot.

## Slide 8
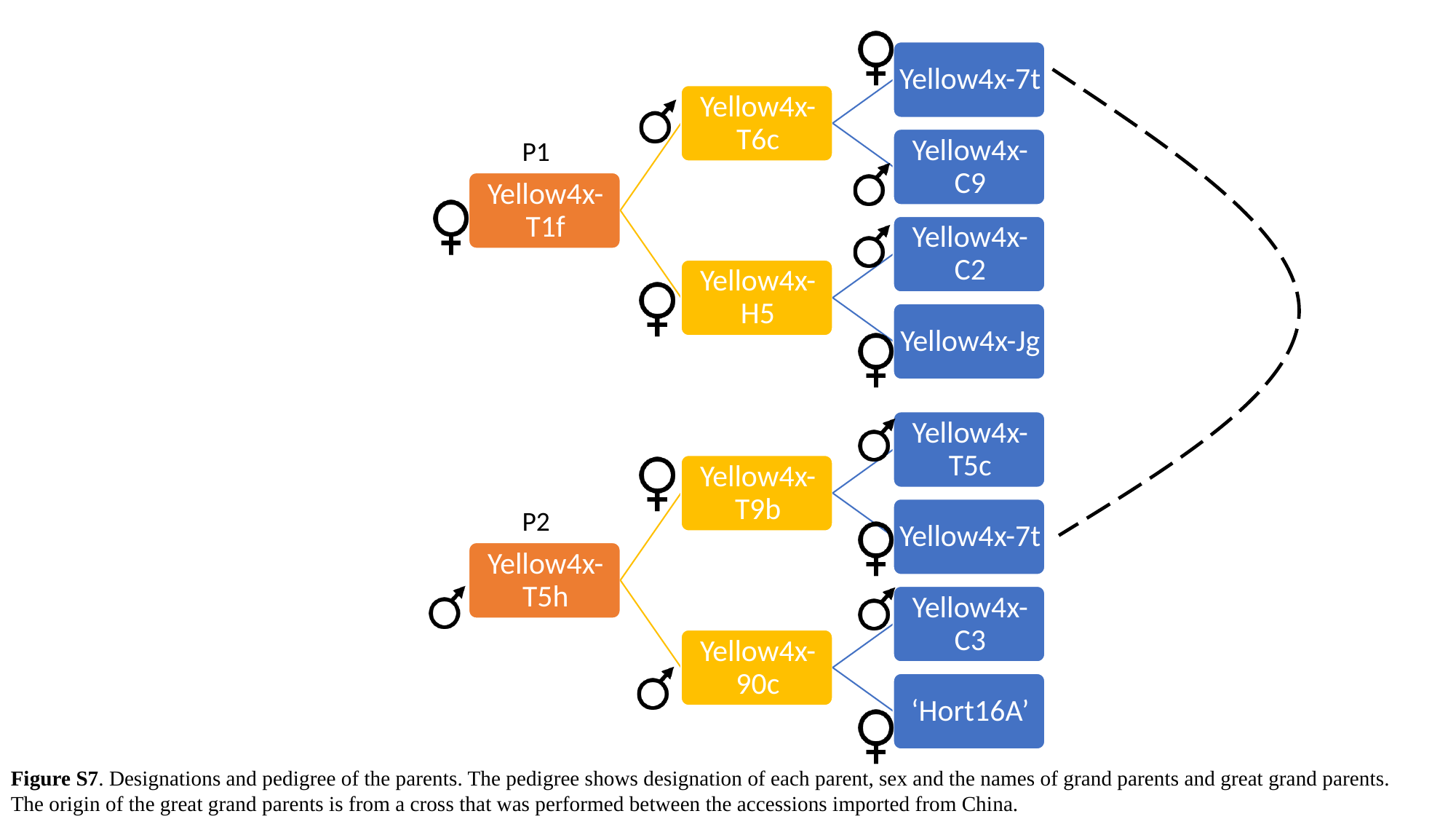

P1
P2
Figure S7. Designations and pedigree of the parents. The pedigree shows designation of each parent, sex and the names of grand parents and great grand parents. The origin of the great grand parents is from a cross that was performed between the accessions imported from China.
